# TIR1-produced cAMP as a second messenger in transcriptional auxin signalling

**DOI:** 10.1038/s41586-025-08669-w

**Published:** 2025-03-05

**Authors:** Huihuang Chen, Linlin Qi, Minxia Zou, Mengting Lu, Mateusz Kwiatkowski, Yuanrong Pei, Krzysztof Jaworski, Jiří Friml

**Affiliations:** 1 Institute of Science and Technology Austria, Klosterneuburg, Austria; 2 Faculty of Synthetic Biology, Shenzhen University of Advanced Technology, Shenzhen, China; 3 Institute of Emerging Agricultural Technology, Shenzhen University of Advanced Technology, Shenzhen, China; 4 Key Laboratory of Quantitative Synthetic Biology, Shenzhen Institute of Synthetic Biology, Shenzhen Institutes of Advanced Technology, Chinese Academy of Sciences, Shenzhen, China; 5 Department of Plant Physiology and Biotechnology, Nicolaus Copernicus University in Toruń, Toruń, Poland

**Keywords:** Auxin, Plant signalling

## Abstract

The phytohormone auxin (Aux) is a principal endogenous developmental signal in plants. It mediates transcriptional reprogramming by a well-established canonical signalling mechanism. TIR1/AFB auxin receptors are F-box subunits of an ubiquitin ligase complex; after auxin perception, they associate with Aux/IAA transcriptional repressors and ubiquitinate them for degradation, thus enabling the activation of auxin response factor (ARF) transcription factors^1–3^. Here we revise this paradigm by showing that without TIR1 adenylate cyclase (AC) activity^4^, auxin-induced degradation of Aux/IAAs is not sufficient to mediate the transcriptional auxin response. Abolishing the TIR1 AC activity does not affect auxin-induced degradation of Aux/IAAs but renders TIR1 non-functional in mediating transcriptional reprogramming and auxin-regulated development, including shoot, root, root hair growth and lateral root formation. Transgenic plants show that local cAMP production in the vicinity of the Aux/IAA–ARF complex by unrelated AC enzymes bypasses the need for auxin perception and is sufficient to induce ARF-mediated transcription. These discoveries revise the canonical model of auxin signalling and establish TIR1/AFB-produced cAMP as a second messenger essential for transcriptional reprograming.

## Abstract (web summary)

cAMP produced by the TIR1/AFB receptors of the main endogenous developmental plant hormone auxin acts as a true second messenger, revising the established paradigm of transcriptional auxin signalling.

## Main

The phytohormone auxin affects almost all aspects of plant life from embryonic to reproductive development^5^. Thus, studies on auxin signalling have always been at the forefront of plant research and have often paved the way for understanding other signalling pathways^6,7^. A main breakthrough came with the identification of the canonical, nuclear pathway involving TIR1 (transport inhibitor response 1)/AFB (auxin signalling F-box protein) auxin receptors and Aux (auxin)/IAA (indole-3-acetic acid) co-receptors acting at the same time as repressors for ARF transcriptional regulators^8^. Although other auxin perception mechanisms exist, including the recently revived cell-surface pathway mediated by the auxin receptor ABP1 (auxin binding protein 1)^9^, as well as direct binding of auxin to transcriptional regulators^10^ and cell cycle regulators^11^, most auxin developmental roles have been attributed to the TIR1 pathway and its role in transcriptional reprogramming.

To regulate transcription, auxin binds to TIR1/AFB receptors and stabilizes their interaction with Aux/IAA repressors, leading to their ubiquitination and degradation. This removal of Aux/IAAs is believed to be the key mechanism relieving ARFs from repression by Aux/IAAs^12^. Different combinations of TIR1/AFBs, Aux/IAAs and ARFs in different cells, and their different affinities and half-lives, enable a rich variety of context-specific transcriptional responses^13^. This textbook model of canonical auxin signalling remained unmodified for almost two decades and does not assume the involvement of second messengers, which are otherwise well known from signalling pathways in animals^14^. Recent unexpected discoveries showed that TIR1/AFB auxin receptors have adenylate cyclase (AC) activity, thus generating cAMP after auxin binding. This AC activity seems important for some auxin functions, mainly auxin-induced root growth inhibition and gravitropism^4^. However, as knowledge of its mechanism has been considered complete, there is no obvious role for the cAMP in the canonical auxin signalling.

Here we revise the established paradigm of transcriptional auxin signalling by showing that, TIR1 AC activity is essential, whereas auxin-induced degradation of Aux/IAAs is not sufficient for mediating downstream transcriptional responses and developmental roles.

## TIR1 AC activity and Aux/IAA degradation

The ubiquitin ligase activity of TIR1/AFBs mediates the degradation of Aux/IAAs^1^. To clarify the role of TIR1 AC activity in downstream auxin signalling, we used TIR1 AC mutant variants (TIR1^ACm1^ and TIR1^ACm3^), which abolish AC activity, and introduced them into the cvxIAA/ccvTIR1 system^4^. The auxin analogue cvxIAA binds and activates only ccvTIR1 but not the endogenous TIR1 or AFBs, thus allowing activation of ccvTIR1 specifically^15^. We generated the mutated *pTIR1::ccvTIR1*^*ACm1*^, *pTIR1::ccvTIR1*^*ACm3*^ and control *pTIR1::ccvTIR1* plants. In addition, we also introduced *pTIR1::TIR1*^*ACm1*^, *pTIR1::TIR1*^*ACm3*^ and *pTIR1::TIR1* into the *tir1-1 afb2-3* (*tir1 afb2*) mutant background^4^.

**Extended Data Fig. 1:**
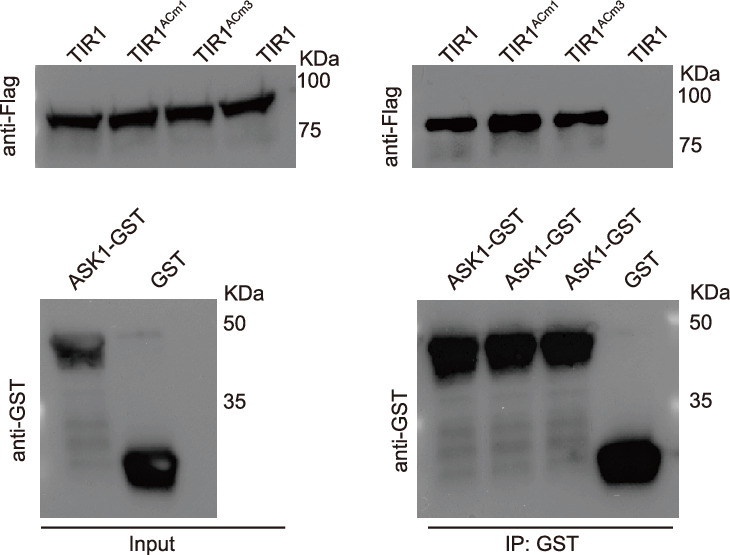
Different TIR1 AC variants interact similarly with ASK1. Pull-down assays were performed with the indicated TIR1-Flag variants translated in vitro using wheat germ extract, and GST-ASK1 purified from *E. coli*. The results shown are from individual gels. 2 independent technically replicates showed the similar results. Source Data

We had previously demonstrated that the TIR1^ACm1^ and TIR1^ACm3^ mutations do not affect auxin perception ability as monitored by auxin-induced interaction between TIR1 and Aux/IAAs^4^. Here we further tested whether these mutations affect the assembly of the SCF^TIR1^ complex by testing the direct interaction of TIR1 with the ASK1 subunit. A pull-down experiment using in vitro translated TIR1-Flag and the mutated variants and glutathione-S-transferase (GST)-ASK1 purified from *Escherichia* *coli* showed that TIR1^ACm1^ and TIR1^ACm3^ do not affect the TIR1 interaction with ASK1 (Extended Data Fig. 1), indicating a correct assembly of the SCF^TIR1^ complex.

**Fig. 1:**
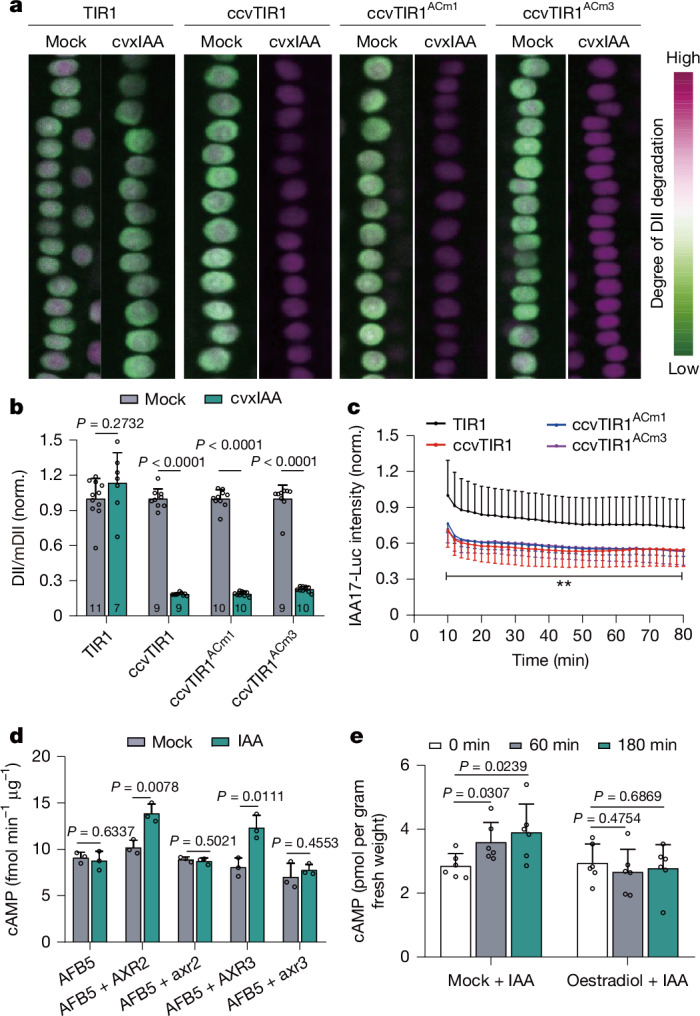
TIR1 AC activity is not required for auxin-induced Aux/IAA degradation. **a**, R2D2 is degraded regardless of TIR1 AC activity. F1 seedlings of the R2D2 reporter crossed with *pTIR1::TIR1*, *pTIR1::ccvTIR1*, *pTIR1::ccvTIR1*^*ACm1*^ and *pTIR1::ccvTIR1*^*ACm3*^ were treated with 200 nM cvxIAA for 1 h. Shown are representative merged images of DII and mDII channels. Original images are in Extended Data Fig. 2a. Two independent technically replicates with similar results. **b**, Quantification of **a** determined as the ratio of DII to mDII. All values were normalized (norm.) to their respective Mock condition (half-strength Murashige and Skoog liquid medium containing an equal concentration of the solvent); mean + s.d., *t*-test, one-sided. The *n* value of roots are indicated within the bars. Two independent technically replicates with similar results. **c**, IAA17-Luc is degraded regardless of TIR1 AC activity. *UBQ10::IAA17-Luc* was crossed into the indicated backgrounds. Data represent a timecourse of residual IAA17-Luc after treatment with 200 nM cvxIAA. Mean ± s.d., *n* = 10 (TIR1), *n* = 12 (ccvTIR1), *n* = 11 (ccvTIR1^ACm1^) and *n* = 10 (ccvTIR1^ACm3^) roots. Asterisks indicate significant differences between TIR1 and ccvTIR1, ccvTIR1^ACm1^ and ccvTIR1^ACm3^. One-way analysis of variance (ANOVA), *P* ≤ 0.01. Two independent technically replicates with similar results. **d**, Nondegradable Aux/IAA proteins (axr2 and axr3) fail to enhance auxin-induced AFB5 AC activity in vitro. cAMP quantification by liquid chromatography with tandem mass spectrometry were conducted in the reaction presence of 10 μM IAA, AXR2/IAA7, AXR3/IAA17, axr2 and axr3 in the indicated combinations. Mean + s.d. from three biological replicates, *t*-test, one-sided. **e**, Expression of stabilized axr3 prevents auxin-induced cAMP production in vivo. cAMP quantification in the *XVE*>*>axr3-mCherry* line. Seedlings were pre-treated with Mock or β-oestradiol for 4 h, followed by treatment with same medium containing 100 nM IAA for different time periods. Mean + s.d. from six biological replicates, *t*-test, one-sided. Source Data

**Extended Data Fig. 2:**
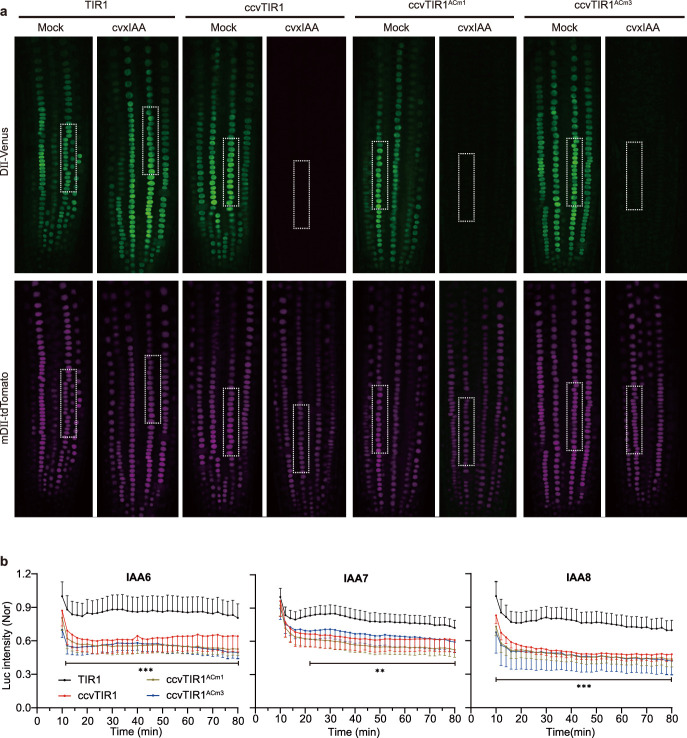
Loss of TIR1 AC activity does not affect the auxin-induced degradation of Aux/IAAs. **a**. The representative images of the two separate channels showing the cvxIAA-induced DII degradation in different genetic background. F1 seedlings were treated with 200 nM cvxIAA for 1 h and the images were taken. Merged images of both channels for DII and mDII from the indicated dashed boxes are showed in Fig. 1a. 2 independent technically replicates with similar results. **b**. The degradation dynamics of different *UBQ10::Aux/IAA-Luc* reporters. Three Aux/IAA-Luc reporter lines were respectively crossed into the indicated genetic background. The time course of the residual Aux/IAA-Luc intensity in the indicated F1 seedlings on medium containing 200 nM cvxIAA is shown. Data are presented as Mean +/− SD, n = 10 (TIR1-IAA6), n = 11 (ccvTIR1-IAA6), n = 11 (ccvTIR1^ACm1^-IAA6), and n = 9 (ccvTIR1^ACm3^-IAA6); n = 9 (TIR1-IAA7), n = 10 (ccvTIR1-IAA7), n = 10 (ccvTIR1^ACm1^-IAA7), and n = 9 (ccvTIR1^ACm3^-IAA7); n = 10 (TIR1-IAA8), n = 9 (ccvTIR1-IAA8), n = 9 (ccvTIR1^ACm1^-IAA8), and n = 8 (ccvTIR1^ACm3^-IAA8) roots. One-way ANOVA, ** p ≤ 0.01, *** p ≤ 0.001. 2 independent technically replicates showed the similar results. Source Data

**Extended Data Fig. 3:**
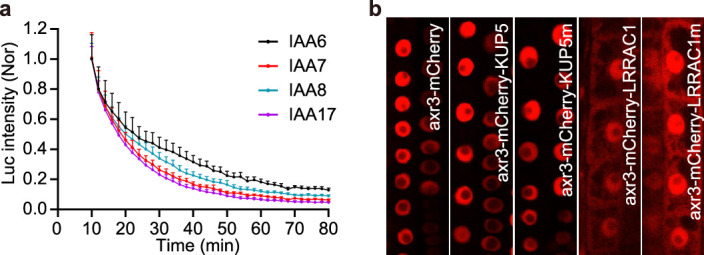
Lability of Aux/IAAs and the induction of the stabilized axr3 variants. **a**. The degradation dynamics of Aux/IAAs in different *UBQ10::Aux/IAA-Luc* lines as indicated. Seedlings were transferred onto the ½ MS medium containing 200 μg/mL cycloheximide. Time interval = 2 min. Mean + SD, n = 10 (IAA6), n = 12 (IAA7), n = 12 (IAA8), and n = 13 (IAA17) roots. 2 independent technically replicates showed the similar results. **b**. Induced expression of XVE>>axr3-mCherry and axr3 fused with different AC enzymes as indicated. Shown are confocal images of mCherry channel in the root tips of *XVE*>*>axr3-mCherry*, *XVE*>*>axr3-mCherry-KUP5*, *XVE*>*>axr3-mCherry-KUP5m*, *XVE*>*>axr3-mCherry-LRRAC1*, and *XVE*>*>axr3-mCherry-LRRAC1m* transgenic seedlings after induction with 1 μM β-estradiol for 4 h. Source Data

Next, we addressed whether the TIR1 AC activity is required for auxin-induced degradation of Aux/IAAs. We crossed the R2D2 ratiometric reporter^16^ for degradation of Aux/IAA into the *ccvTIR1* transgenic lines. cvxIAA treatment had no effect in *pTIR1::TIR1*, but caused specific removal of DII signal in the *pTIR1::ccvTIR1* line. Notably, cvxIAA-triggered DII degradation occurred identically in the *pTIR1::ccvTIR1*^*ACm1*^ and *pTIR1::ccvTIR1*^*ACm3*^ lines (Fig. 1a,b and Extended Data Fig. 2a). We also crossed several *UBQ10::IAAs-luciferase* lines expressing different full-length Aux/IAAs, including IAA6, IAA7, IAA8 and IAA17 (refs. ^17–19^) (Extended Data Fig. 3a), into the ccvTIR1 transgenic lines, and observed comparable cvxIAA-induced degradation of all these Aux/IAAs in *pTIR1::ccvTIR1*, *pTIR1::ccvTIR1*^*ACm1*^ and *pTIR1::ccvTIR1*^*ACm3*^ (Fig. 1c and Extended Data Fig. 2b). Thus, consistent with TIR1^ACm1^ and TIR1^ACm3^ mutations not affecting auxin-induced TIR1–Aux/IAA interaction^4^ and SCF^TIR1^ complex assembly, these observations show that loss of TIR1 AC activity does not affect auxin-induced degradation of Aux/IAAs.

This shows that the AC activity of TIR1/AFBs is not required for auxin-induced degradation of Aux/IAAs, meaning that TIR1/AFBs AC and SCF^TIR1^ E3 ligase activities are independent.

## Aux/IAA stabilizing mutants and cAMP production

In a complementary experiment, we tested the effect of degradation of Aux/IAA on AC activity. Our previous results showed that auxin-induced assembly of the TIR1–Aux/IAA complex enhances AC activity^4^. To elaborate on these observations, we expressed and purified AFB5, the co-receptors AXR2 and AXR3 (also known as IAA7 and IAA17), and their axr2 and axr3 variants carrying point mutations in Domain II (DII), which are known to abolish auxin-induced TIR1/AFBs-Aux/IAA interaction and degradation of Aux/IAA^1,2^. In vitro AC activity experiments showed that IAA alone or AXR2 or AXR3 alone have no effect on AC activity. However, IAA together with either AXR2 or AXR3 increased AC activity, whereas combinations with the mutated axr2 or axr3 did not (Fig. 1d).

**Extended Data Fig. 4:**
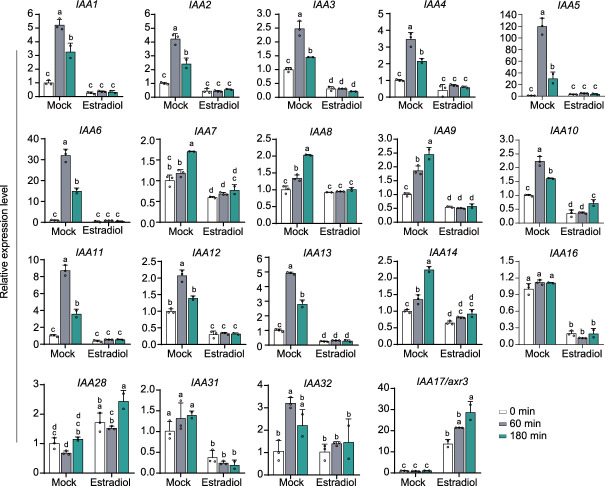
Most *Aux/IAAs* transcripts are downregulated in the *XVE*>*>axr3-mCherry* line. The expression levels of the selected *Aux/IAA* genes before and after auxin treatment were largely reduced in the *XVE*>*>axr3-mCherry* transgenic line induced by β-estradiol. Seedlings were pre-treated with Mock or β-estradiol for 4 h, and were then treated with the same medium containing 100 nM IAA for different time periods. Gene expression level was determined by RT-qPCR. Data are presented as Mean + SD from more than 2 biologically replicates. Two-way ANOVA, p ≤ 0.05. Different lowercase letters indicate significant differences. Source Data

To verify these results in vivo, we generated the inducible *XVE*>*>axr3-mCherry* transgenic line. After induction with β-oestradiol for 4 h, a sufficient amount of axr3-mCherry protein can be detected (Extended Data Fig. 3b). IAA treatment induced cAMP production normally in this line under control conditions, but when axr3 expression was induced by β-oestradiol, auxin-induced cAMP accumulation was abolished (Fig. 1e). Gene expression analysis showed that expression of the stabilized axr3 leads to the transcriptional downregulation of most *Aux/IAA* genes (Extended Data Fig. 4) and also a degradation of DII of Aux/IAA after induction of axr3-mCherry, as monitored by complete loss of the DII-Venus reporter signal^20^. This shows that accumulation of the stabilized axr3 results in the reduction of endogenous Aux/IAA proteins and mRNA in vivo, providing an explanation for why auxin-induced cAMP accumulation is abolished in this line. This further confirms that TIR1/AFB–Aux/IAA interaction is necessary for auxin-induced stimulation of the AC activity of TIR1/AFBs.

Overall, these experiments show that the AC activity of TIR1/AFB is not required for auxin-induced degradation of Aux/IAA, whereas the well-known stabilizing mutations in Aux/IAAs, which prevent their interaction with TIR1/AFBs and reduce the endogenous amounts of other Aux/IAAs in planta, lead to a failure in auxin-induced cAMP production.

## TIR1 AC activity for transcription activation

**Fig. 2:**
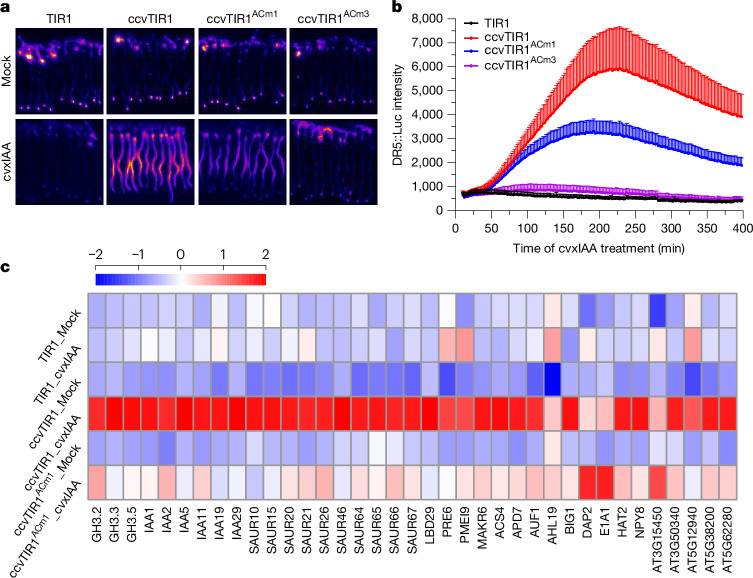
TIR1 AC activity is crucial for the auxin-mediated transcriptional response. **a**, AC-deficient TIR1 fails to mediate auxin-induced activation of *DR5::Luc* reporter. F1 seedlings of *DR5::Luc* cross with *pTIR1::TIR1*, *pTIR1::ccvTIR1*, *pTIR1::ccvTIR1*^*ACm1*^ and *pTIR1::ccvTIR1*^*ACm3*^ were treated with 200 nM cvxIAA. Images showed representative examples of *DR5::Luc* intensity after 4 h. The colour mode of Fire LUT was applied to the images. Quantification of the *DR5::Luc* intensity is showed in Extended Data Fig. 2a. **b**, Timecourse of *DR5::Luc* signal in the indicated backgrounds on medium containing 200 nM cvxIAA. Mean + s.d., *n* = 12 (TIR1), *n* = 10 (ccvTIR1), *n* = 10 (ccvTIR1^ACm1^) and *n* = 12 (ccvTIR1^ACm3^) roots. The statistical analysis is shown in Source Data. Two independent technically replicates showed similar results. **c**, AC-deficient TIR1 fails to mediate global auxin-induced transcriptional reprogramming as determined by RNA-seq in *pTIR1::TIR1*, *pTIR1::ccvTIR1* and *pTIR1::ccvTIR1*^*ACm1*^. Seedlings were treated with 200 nM cvxIAA for 3 h. The differentially expressed genes with log_2_ fold change ≥ 1 and adjusted *P* value ≤ 0.001 between Mock and cvxIAA treatments from three biological replicates were used to create the heatmap. Raw data was normalized using *Z* score scaling. Source Data

**Extended Data Fig. 5:**
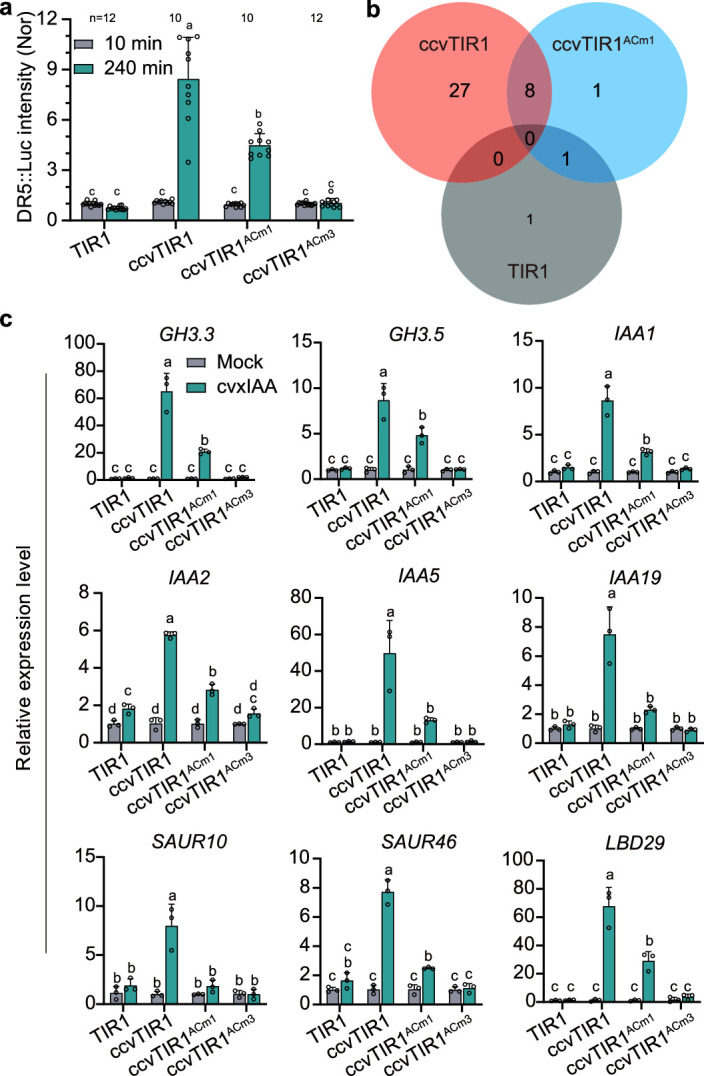
AC activity of TIR1 plays a key role in auxin-induced transcriptional response. **a**. Quantification of *DR5::Luc* intensity in Fig. 2a. All data are normalized to the average of the respective Mock group. Mean + SD, n value is indicated. Two-way ANOVA, p ≤ 0.05. 2 independent technically replicates showed the similar results. **b**. The Venn diagram shows the number of differentially expressed genes with a Log2FC ≥ 1 and p adj ≤ 0.001 in different transgenic lines, comparing Mock to cvxIAA treatment. **c**. Expression levels of the selected auxin-responsive genes in the indicated transgenic lines. Seedlings were treated with 200 nM cvxIAA for 3 h. RT-qPCR was performed with *PP2A* as the internal control. Relative expression values were normalized to the average of the respective Mock group. Mean + SD from 3 biological replicates. Two-way ANOVA, p ≤ 0.05. Different lowercase letters indicate significant differences (**c**). Source Data

Previous observations indicated that cAMP produced by TIR1 AC might be important for auxin-mediated transcription^4^. To further investigate the role of AC activity in regulating TIR1/AFBs-mediated transcription, we introduced the transcriptional auxin signalling output sensor *DR5::Luciferase* (*DR5::Luc*)^21^ into *pTIR1::TIR1, pTIR1::*ccv*TIR1*, *pTIR1::*ccv*TIR1*^*ACm1*^ and *pTIR1::*ccv*TIR1*^*ACm3*^ plants. Our results showed that cvxIAA specifically activated luciferase signal in *pTIR1::*ccv*TIR1* but not in the *pTIR1::TIR1* line. Notably, cvxIAA-induced *DR5::Luc* activation was strongly reduced in *pTIR1::*ccv*TIR1*^*ACm1*^ and completely lost in the *pTIR1::*ccv*TIR1*^*ACm3*^ line (Fig. 2a,b and Extended Data Fig. 5a) indicating that TIR1 AC activity is indispensable for auxin-induced transcription. We also explored the requirement of TIR1 AC activity on global auxin-induced transcriptional reprogramming (Supplementary Information 1). RNA sequencing (RNA-seq)-based transcriptome data in *pTIR1::*ccv*TIR1* showed that cvxIAA treatment induces many early auxin response genes, for example, *GH3s*, *Aux/IAAs* and *SAURs*. However, this global transcriptional induction was strongly attenuated in the *pTIR1::*ccv*TIR1*^*ACm1*^ line, as also confirmed by quantitative PCR with reverse transcription (RT–qPCR)^4^ (Fig. 2c and Extended Data Fig. 5b,c), with the exception of three genes for which we observed activation instead of attenuation in the *pTIR1::*ccv*TIR1*^*ACm1*^ line.

Our findings show that TIR1-mediated cAMP production is indispensable for presumably all downstream transcriptional regulation, including when auxin-induced degradation of Aux/IAA repressors occur normally.

## TIR1 AC activity for auxin-regulated development

We used complemented lines in which *pTIR1::TIR1*, *pTIR1::TIR1*^*ACm1*^ and *pTIR1::TIR1*^*ACm3*^ were introduced into the *tir1 afb2* mutant, as well as *pTIR1::ccvTIR1*, *pTIR1::*ccv*TIR1*^*ACm1*^ and *pTIR1::*ccv*TIR1*^*ACm3*^ lines to investigate the importance of TIR1 AC activity for various aspects of auxin-regulated development.

**Extended Data Fig. 6:**
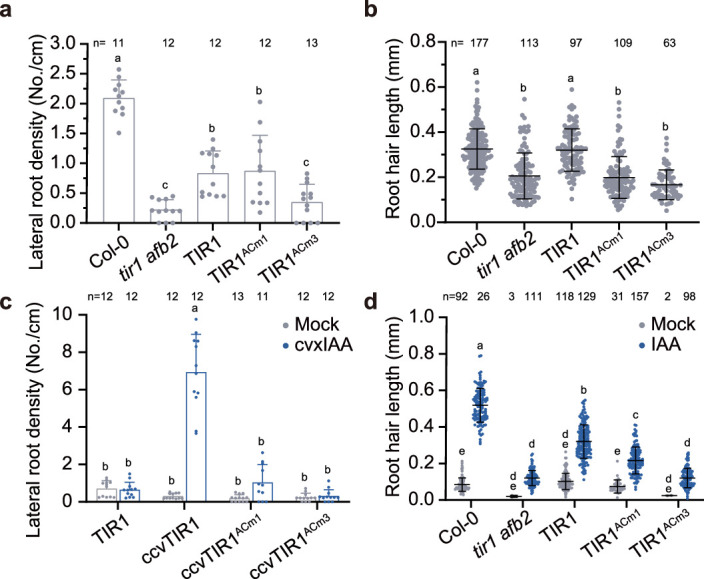
Crucial roles of TIR1 AC activity in auxin-regulated development. **a**. Quantification of lateral root density of the indicated 10-day-old seedlings. Mean + SD, n value of roots are indicated. One-way ANOVA, p ≤ 0.05. **b**. The indicated complementation lines were transferred to new medium for 1.5 days, and root hair length was measured. Mean ± SD, n value of root hairs are indicated. One-way ANOVA, p ≤ 0.05. **c**. Quantification of lateral root density. Seedlings of the indicated genotypes were transferred onto 200 nM cvxIAA-containing medium for 4 days. Mean + SD, n value of roots are indicated. Two-way ANOVA, p ≤ 0.05. **d**. Quantification of root hair length. Seedlings of the indicated genotypes were transferred onto medium with 10 nM IAA for 1.5 days. Mean ± SD, n value of root hairs are indicated. Two-way ANOVA, p ≤ 0.05. 2 independent technically replicates showed the similar results. Different lowercase letters indicate significant differences. Source Data

First, we found that *pTIR1::TIR1*^*ACm3*^ failed to complement the lateral root formation defects of *tir1 afb2* (Extended Data Fig. 6a). Similarly, neither *pTIR1::TIR1*^*ACm1*^ nor *pTIR1::TIR1*^*ACm3*^ rescue the root hair growth defects of *tir1 afb2* (Extended Data Fig. 6b), consistent with their inability to rescue the defect in gravitropic response^4^, collectively indicating that TIR1 AC activity is required for normal auxin signalling-mediated development.

**Fig. 3:**
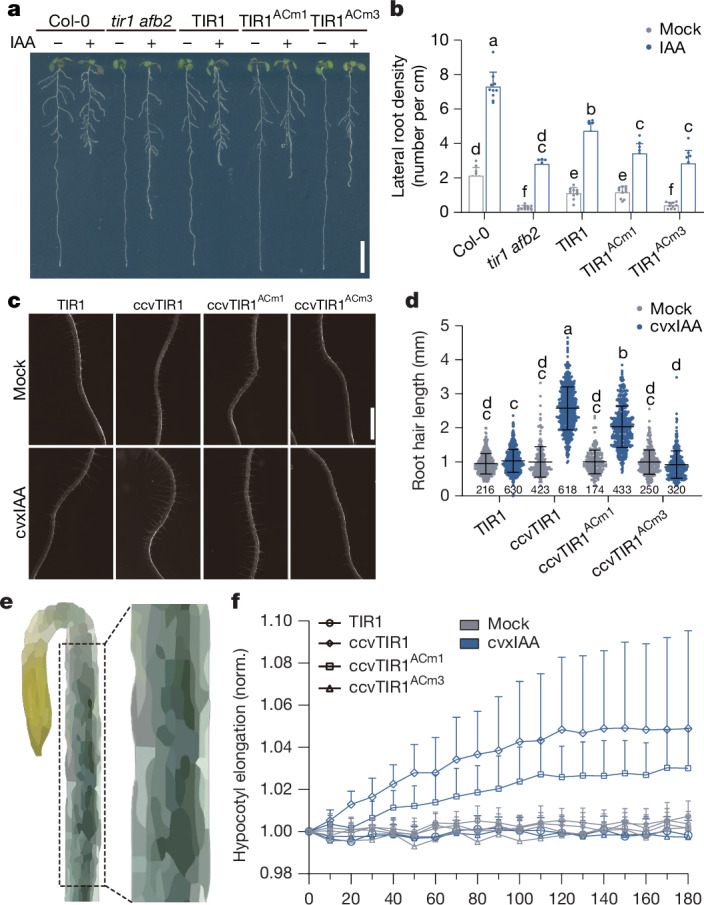
TIR1 AC activity is crucial for auxin-regulated lateral root formation, root hair growth and hypocotyl elongation. **a**, Lateral root formation. *pTIR1-TIR1, pTIR1-TIR1*^*ACm1*^ and *pTIR1-TIR1*^*ACm3*^ seedlings were transferred for 4 days onto medium containing 100 nM IAA. **b**, Quantification of lateral root density (lateral root number per main root length) of roots shown in **a**. Mean + s.d., *n* = 10 roots. Two-way ANOVA, *P* ≤ 0.05. **c**, Root hair elongation. Representative images show root hairs of different genotypes grown on Mock or 100 nM cvxIAA for 1.5 days. **d**, Quantification of the root hair length of roots shown in **c**. Mean ± s.d., *n* values of root hairs are indicated below plots. Two-way ANOVA, *P* ≤ 0.05. **e**, Hypocotyl segments elongation. Schematic diagram illustrating the source of hypocotyl segments from etiolated seedlings used for the auxin-induced hypocotyl elongation assay. **f**, Elongation kinetics of hypocotyl segments. The segments were transferred to Mock or 1 µM cvxIAA; the images were taken with an interval of 10 min. Mean + s.d., *n* = 11 (TIR1-Mock), *n* = 9 (TIR1-cvxIAA), *n* = 12 (ccvTIR1-Mock and cvxIAA), *n* = 13 (ccvTIR1^ACm1^-Mock), *n* = 12 (ccvTIR1^ACm1^-cvxIAA), *n* = 14 (ccvTIR1^ACm3^-Mock) and *n* = 13 (ccvTIR1^ACm3^-cvxIAA) hypocotyl segments. The statistical analysis is showed in the Source Data. Two independent technically replicates showed similar results. Scale bars, 1 cm (**a**), 1 mm (**c**). Lowercase letters above data points (**b**,**d**) indicate significant differences. Source Data

We then assessed the auxin effect on lateral root formation. IAA induces lateral root formation in wild type, whereas the *tir1 afb2* was almost insensitive—a defect that was rescued by *pTIR1::TIR1*, only marginally by *pTIR1::TIR1*^*ACm1*^ and not at all by *pTIR1::TIR1*^*ACm3*^ (Fig. 3a,b). Similarly, cvxIAA-induced lateral root formation in *pTIR1::ccvTIR1*, but not in *pTIR1::*ccv*TIR1*^*ACm3*^, whereas *pTIR1::*ccv*TIR1*^*ACm1*^ showed a weak response (Extended Data Fig. 6c). This result shows that TIR1 AC activity is required for auxin-induced lateral root formation.

We also tested whether TIR1 AC activity is necessary for auxin-induced root hair growth by transferring the complemented lines onto medium containing IAA. IAA promoted root hair growth in *tir1 afb2* only slightly, but triggered strong root hair growth in the *pTIR1::TIR1* but not *pTIR1::TIR1*^*ACm1*^ and *pTIR1::TIR1*^*ACm3*^ lines (Extended Data Fig. 6d). *pTIR1::ccvTIR1* responded normally to cvxIAA treatment, whereas *pTIR1::ccvTIR1*^*ACm1*^ was defective in this response and *pTIR1::ccvTIR1*^*ACm3*^ completely insensitive (Fig. 3c,d). This shows that TIR1 AC activity is also essential for auxin effect on promoting root hair growth.

The induction of hypocotyl elongation (Fig. 3e) is another typical auxin response. Hypocotyl segments will elongate significantly when transferred to medium containing auxin^22,23^. The cvxIAA treatment promoted hypocotyl elongation in the *pTIR1::ccvTIR1* line but not in the *pTIR1::ccvTIR1*^*ACm3*^, whereas *pTIR1::ccvTIR1*^*ACm1*^ is only partly responsive (Fig. 3f). This indicates that TIR1 AC activity is crucial also for auxin-induced hypocotyl elongation.

These experiments collectively show that TIR1 AC activity is required not only for the auxin-induced root growth inhibition and gravitropism^4^ but also for lateral root formation, root hair growth and hypocotyl elongation; all typical responses mediated by the transcriptional auxin signalling. Thus, presumably the TIR1 AC activity is essential for all transcriptional developmental roles of TIR1/AFBs.

## cAMP production for transcription and development

The strong developmental phenotypes and defective transcriptional regulation in *Arabidopsis* lines with abolished TIR1 AC activity but a normal auxin-induced degradation of Aux/IAAs is not aligned with a current model of canonical auxin signalling and advocates for cAMP acting as an essential second messenger having target and regulating transcription downstream of TIR1/AFBs.

**Fig. 4:**
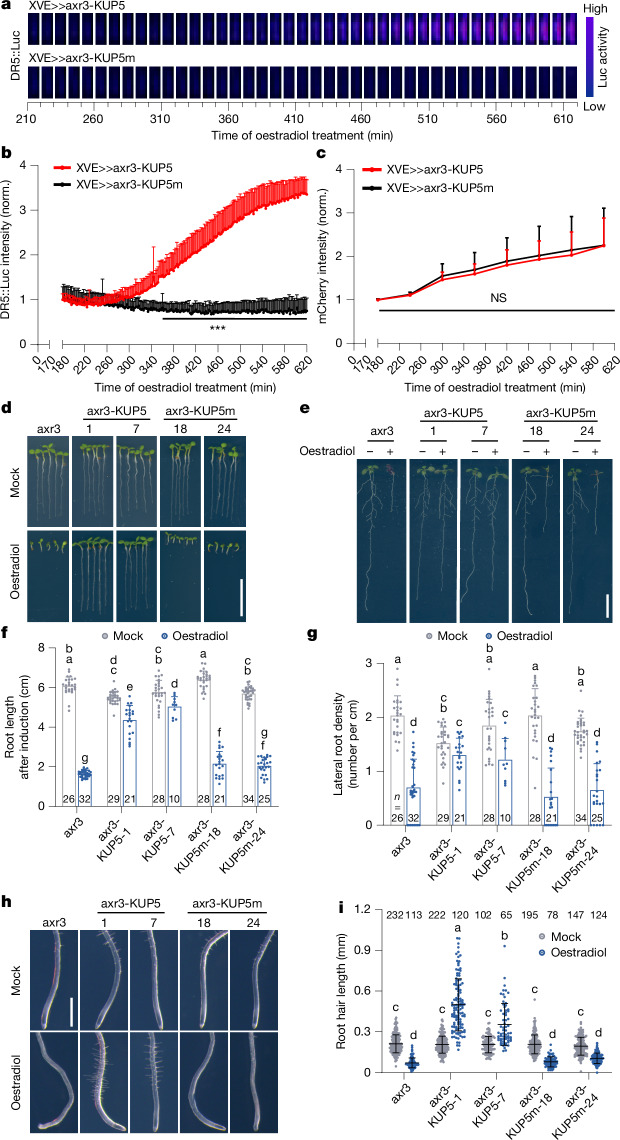
Auxin-independent cAMP production activates transcriptional auxin responses. **a**,**b**, Axr3-KUP5 AC activity induces *DR5::Luc* expression. Montage of false colour images show *DR5::Luc* activity in the root tip of *XVE*>*>axr3-mCherry-KUP5* (axr3-KUP5) or *XVE*>*>axr3-mCherry-KUP5m* (axr3-KUP5m) after 1 μM β-oestradiol treatment. Frames represent 10-min intervals (**a**), while quantification uses 2-min intervals (**b**). Significant differences (*t*-test, one-sided, *P* ≤ 0.001) emerged from 348 min. Mean + s.d., *n* = 11 (axr3-KUP5) and *n* = 11 (axr3-KUP5m) roots (**b**). All data were normalized to the respective averages at 180 min. **c**, Comparable axr3-KUP5 and axr3-KUP5m accumulation after induction. The fluorescence intensity of mCherry was measured starting from 180 min. All data were normalized to the respective averages at 180 min. Mean + s.d., *n* = 7 (axr3-KUP5) and *n* = 8 (axr3-KUP5m) roots; *t*-test, one-sided, non-significant (NS), *P* > 0.5. **d**, axr3-KUP5 rescues the rootless defect caused by the overexpressed axr3 or axr3-KUP5m. Images of 5-day-old seedlings; *XVE*>*>axr3* (axr3), *axr3-KUP5* and *axr3-KUP5m* transgenic seeds (1, 7, 18 and 24) were germinated on Mock or 1 µM β-oestradiol-containing medium. **e**–**g**, axr3-KUP5 promotes lateral root formation. Representative images (**e**) and quantitative data of root length (**f**) and lateral root density (**g**) are shown. The 5-day-old seedlings described in **d** were transferred to Mock or 1 µM β-oestradiol-containing medium for a further 4 days. Mean + s.d., the *n* value of roots in **f** and **g** are indicated within the bars. Two-way ANOVA, *P* ≤ 0.05 (**f**,**g**). **h**,**i**, axr3-KUP5 promotes root hair elongation. Representative images (**h**) and quantitative root hair length data (**i**) are shown. The 5-day-old seedlings described in **d** were transferred to Mock or 1 µM β-oestradiol-containing medium for a further 2 days. Mean ± s.d., the *n* value of root hairs in **i** are indicated above the plots. Two-way ANOVA, *P* ≤ 0.05 (**i**). Two independent technically replicates showed the similar results. Scale bars, 1 cm (**d**,**e**), 1 mm (**h**). Lowercase letters above data points (**f**,**g**,**i**) indicate significant differences. Source Data

**Extended Data Fig. 7:**
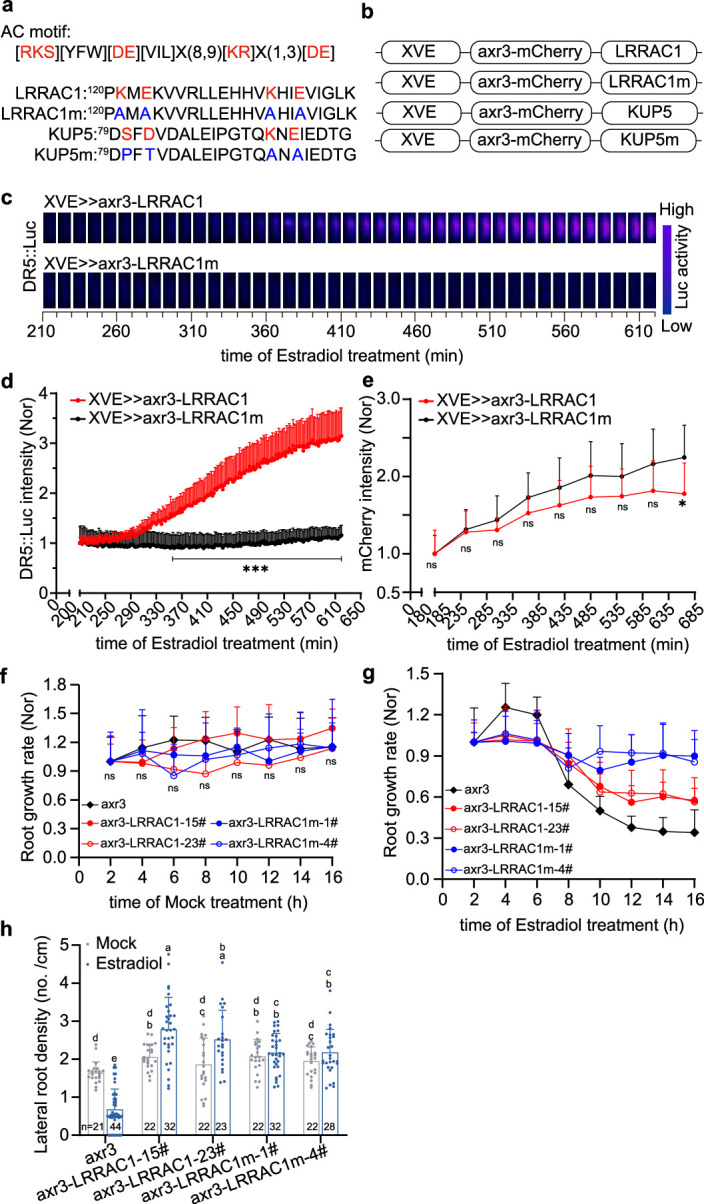
cAMP can bypass Aux/IAA degradation and activate auxin-responsive transcription. **a**. The typical conserved AC motif is shown in the upper part. The sequence of the AC motif from LRRAC1 or KUP5 and its mutated form LRRAC1m and KUP5m is shown below. Red font indicates the core animo acid site. Blue font indicates the mutant form. **b**. The plasmid structure showing the fusion of the AC enzyme (KUP5 or KUP5m or LRRAC1 or LRRAC1m) to stabilized axr3-mCherry driven by the β-estradiol-inducible promoter. **c. d**. Induction of axr3-LRRAC1 activates *DR5::Luc* expression. Montage of false colour images show *DR5::Luc* activity in the root tip of *DR5::Luc* signal in root tips of *XVE*>*>axr3-mCherry-LRRAC1* (axr3-LRRAC1) or -*LRRAC1m* (axr3-LRRAC1m) in the *DR5::Luc* background after treatment with 1 μM β-estradiol. Frames represent 10 min interval **(c)**. Quantification data are shown with a time interval of 2 min. All data were normalized to the average of the respective values at 210 min. Significant differences (*** p ≤ 0.001) were observed starting from 340 min. Mean + SD, n = 14 (axr3-LRRAC1) and n = 17 (axr3-LRRAC1m) roots, *t* test, one-sided **(d)**. **e**. Similar accumulation of axr3-LRRAC1 and axr3-LRRAC1m after induction with 1 μM β-estradiol. The normalized fluorescence intensity of mCherry in the indicated transgenic lines is shown at the different time points after β-estradiol treatment. All data are normalized to the intensity at 185 min. Mean + SD, n = 9 roots, *t* test, one-sided. ns p > 0.5, * p ≤ 0.05. **f-h**. The LRRAC1 fused to the stabilized axr3 inhibits primary root elongation rate and promotes lateral root formation. The *XVE*>*>axr3* (axr3), axr3-LRRAC1 and (axr3-LRRAC1m seedlings were transferred to Mock or 1 µM β-estradiol-containing medium, and the root elongation rate was calculated and normalized to the value of the first 2 h. Mean + SD, n = 10 roots **(f)**; n = 10 (axr3, axr3-LRRAC1m-1# and -4#), and n = 9 (axr3-LRRAC1-15# and -23#) roots **(g)**. The statistical analysis for **(f, g)** is included in the **Source Data**. For the quantification of lateral root density (lateral root number/ main root length), *XVE*>*>axr3* (axr3), axr3-LRRAC1 and axr3-LRRAC1m seedlings were transferred onto medium containing 1 µM β-estradiol for 4 days **(h)**. n value is indicated **(h)**. Mean + SD. Two-way ANOVA, p ≤ 0.05 **(h)**. Different lowercase letters indicate significant differences. Source Data

To test this possibility in planta, we engineered independent cAMP production in the context of nuclear auxin signalling. We fused the characterized AC domain from KUP5 or its inactive mutant variant KUP5m to axr3 (ref. ^24^) (Extended Data Fig. 7a,b) and generated *XVE*>*>axr3-mCherry-KUP5* (axr3-KUP5), *XVE*>*>axr3-mCherry-KUP5m* (axr3-KUP5m) and *XVE*>*>axr3-mCherry* (axr3) transgenic plants enabling their β-oestradiol-inducible expression. We also used another characterized AC domain and its inactive form, LRRAC1 and LRRAC1m^14^ and generated similar *XVE*>*>axr3-mCherry-LRRAC1* (axr3-LRRAC1) and *XVE*>*>axr3-mCherry-LRRAC1m* (axr3-LRRAC1m) lines (Extended Data Fig. 7a,b). The constructs have the axr3 mutation in the DII preventing interaction with TIR1/AFBs but preserving interaction with ARFs. Thus, this experiment bypasses the TIR1/AFB activity. When introduced into *DR5::Luc* background, induction of axr3-KUP5 and axr3-LRRAC1 expression, but not their AC-inactive variants, activated the DR5 transcriptional auxin response (Fig. 4a,b and Extended Data Fig. 7c,d). Notably, all axr3 variants were expressed similarly as monitored by the mCherry signal (Fig. 4c and Extended Data Fig. 7e). This shows that fusion of active AC to axr3 is sufficient to activate ARF-driven transcription. Presumably, the DR5 activity is a result of a balance between activation by the locally produced cAMP and repression by Aux/IAAs and their interactor TOPLESS^7^.

Next, we tested developmental consequences of the auxin-independent cAMP production. Germination of axr3 or axr3-KUP5m seedlings on β-oestradiol-containing plates completely blocked root formation, whereas axr3-KUP5 lines formed well-developed roots (Fig. 4d). When 4-day-old seedlings were transferred on medium containing β-oestradiol, axr3 or axr3-KUP5m drastically inhibited root growth (Fig. 4e,f), lateral root development (Fig. 4g) and root hair growth (Fig. 4h,i), whereas axr3-KUP5 recovered root growth (Fig. 4e,f), maintained lateral root development (Fig. 4e,g) and promoted root hair growth even further (Fig. 4h,i). Thus, fusion of functional KUP5-based AC motif with axr3 counteracted its inhibitory effect on auxin-regulated development.

The LRRAC1 and LRRAC1m fusions affected the nuclear localization of axr3, making it partially cytosolic (Extended Data Fig. 3b) and attenuating its inhibitory effect^20^. Accordingly, induction of axr3 expression inhibited root growth, whereas axr3-LRRAC1m lost this effect, but axr3-LRRAC1 reduced the root growth rate again (Extended Data Fig. 7f,g), indicating reactivation of the auxin response. This is even more obvious in the case of lateral root development: axr3 expression inhibited lateral root formation, axr3-LRRAC1m no longer had such effect, but axr3-LRRAC1 further enhanced lateral root development; all effects similar to auxin treatment (Extended Data Fig. 7h). Thus, fusion of the LRRAC1-based AC motif with axr3 mimics the effects of auxin on root growth and lateral root formation.

Thus, when active AC enzymes are expressed in the vicinity of transcriptional auxin signalling components, they activate transcriptional auxin response and mimic auxin effects on development, whereas mutated, inactive AC controls do not have these effects. Together, these results demonstrate that local cAMP production in the context of auxin signalling can bypass the need of auxin perception and degradation of Aux/IAAs and is sufficient to mediate transcriptional output of auxin signalling.

## Discussion

Elucidation of canonical, TIR1 receptor-mediated auxin signalling mechanism represented a milestone in understanding of signalling in plants^1–3^. Its key step—the SCF^TIR1^ E3 ubiquitin ligase-mediated ubiquitination and subsequent degradation of transcriptional repressors—became a blueprint for discoveries of similar mechanisms in other prominent signalling pathways^25,26^. It withstood the test of time and remained unmodified for two decades. Therefore, the recent discovery of AC activity conserved in all TIR1/AFB auxin receptors was unexpected^4^, not least because production of cAMP as another output of auxin perception (next to the ubiquitination of Aux/IAAs) does not fit the existing paradigm and a role of cAMP as a second messenger in plants is still controversial.

**Extended Data Fig. 8:**
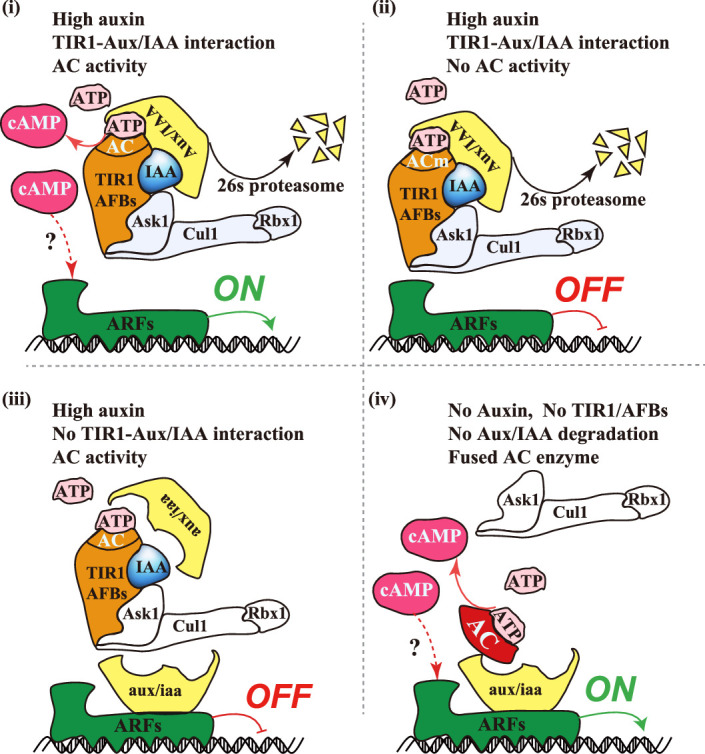
A simplified scheme summarizing the novel insights for auxin signalling pathway. **(i)** Auxin induces Aux/IAA degradation and cAMP production in parallel, resulting in transcriptional auxin responses in undisturbed situation. **(ii)** When AC activity of TIR1/AFBs is eliminated (ACm), auxin normally induces the degradation of Aux/IAAs but cannot activate ARF-dependent transcription. **(iii)** Interaction between TIR1/AFBs and Aux/IAAs is necessary for both Aux/IAA degradation and cAMP production. Stable aux/iaa, cannot interact with TIR1/AFBs, and prevent cAMP production. **(iv)** Local production of cAMP at the Aux/IAA-ARF regulatory niche bypasses auxin perception, Aux/IAAs’ degradation and activates ARF-mediated transcription.

Here we assessed the relevance of TIR1/AFBs AC activity in canonical auxin signalling (Extended Data Fig. 8). We used transgenic lines expressing mutated versions of TIR1 receptor lacking AC activity^4^. They were either introduced into *tir1 afb2* mutant or engineered as a ccvTIR1 synthetic biology tool, enabling their specific activation by cvxIAA^15^. In these lines, auxin (1) does not induce cAMP production^4^, (2) normally induces degradation of Aux/IAA repressors (Fig. 1a–c and Extended Data Fig. 2), (3) does not induce transcription (globally, or of selected auxin-inducible genes; Fig. 2 and Extended Data Fig. 5) and (4) cannot regulate developmental processes associated with transcriptional regulation, such as growth and organogenesis (Fig. 3 and Extended Data Fig. 6).

Thus, although auxin induces degradation of Aux/IAAs normally, the downstream transcription is not activated and auxin-regulated development is defective if the TIR1/AFB receptors are unable to produce cAMP. This revises the accepted notion that degradation of Aux/IAA repressors downstream of TIR/AFBs-mediated auxin perception is the key step in ARF activation to mediate transcription of auxin-inducible genes. It shows that cAMP production is essential, whereas degradation of Aux/IAAs is not sufficient for the transcriptional signalling (Fig. 4 and Extended Data Fig. 7).

In contrast, cAMP production seems sufficient to activate transcription of auxin-regulated genes. When cAMP is produced in the vicinity of the Aux/IAA–ARF complex by inducible expression of previously characterized ACs^14,24^ fused to the ARF interactor axr3, this was sufficient to activate transcriptional auxin response reporter DR5. Two different unrelated ACs (KUP5 and LRRAC1) had the same effect, whereas their AC-inactive versions were ineffective (Fig. 4a,b and Extended Data Fig. 7c,d). The DR5 activation in this experimental set-up occurred in the presence of otherwise repressing axr3 protein. Accordingly, expression of axr3-AC lines showed ‘auxin-like’ phenotypes in growth and organogenesis regulation (Fig. 4d–i and Extended Data Fig. 7f–h). This indicates that cAMP produced locally in the vicinity of ARF–Aux/IAA complex can bypass auxin perception, overcome the repressive effect of Aux/IAAs and activate ARFs to mediate transcription. This supports that cAMP production is not only required but also sufficient to activate ARF-mediated transcription.

The previously assumed crucial role of degradation of Aux/IAAs in auxin signalling was supported by the identification of mutations in DII of Aux/IAA repressors that abolish their interaction with TIR1/AFBs and render them stable. Such mutants show auxin-resistant phenotypes and strong developmental defects^1,2^. However, those mutated Aux/IAA proteins also interfere with auxin-induced cAMP production in vitro and in vivo (Fig. 1d,e). Thus, defects in these mutants can arise from both over-accumulation of stabilized Aux/IAAs and/or lack of auxin-induced cAMP production.

The role of Aux/IAAs degradation downstream of auxin perception remains unclear. It certainly can serve as a negative feedback mechanism stopping TIR1/AFBs-mediated cAMP production since Aux/IAAs are required for auxin-induced stimulation of TIR1/AFB AC activities (Fig. 1d,e). As expansion of the whole Aux/IAA family in seed plants indicates, it will have other roles, presumably in providing prolonged derepression of ARFs or conveying specificity in transcriptional response in different contexts.

These findings also demonstrate that cAMP acts in the context of transcriptional auxin signalling as a true second messenger. It is produced by TIR1/AFB receptors but has a target beyond. The obvious candidates for cAMP targets here are ARF or Aux/IAA proteins but which is targetted and how cAMP modifies their activity remains to be seen.

## Methods

### Plant materials and growth conditions

All the *Arabidopsis* transgenic lines and mutants, were in the Columbia-0 (Col-0) background except R2D2, which was in the Columbia-Utrecht (Col-utr) ecotype. The *tir1-1 afb2-3* mutant^15^, R2D2 ratiometric reporter line^16^, *UBQ10::IAA6-Luc*^18^, *UBQ10::IAA7-Luc*, *UBQ10::IAA8-Luc*^17^ and *UBQ10::IAA17-Luc*^19^ reporters and the *DR5::Luc* reporter^21^ have all been described previously. The complementation lines *pTIR1::TIR1*, *pTIR1::TIR1*^*ACm1*^ and *pTIR1::TIR1*^*ACm3*^ in *tir1-1 afb2-3*, as well as the *pTIR1::ccvTIR1*, *pTIR1::ccvTIR1*^*ACm1*^ and *pTIR1::ccvTIR1*^*ACm3*^ engineering lines in *tir1-1 afb2-3* were published recently^4^.

To generate the *XVE*>*>axr3-mCherry* transgenic line, the P88L mutation was introduced directly into AXR3(IAA17). DNA fragments encoding axr3 and mcherry with more than 15-bp overlap sequences were then assembled into the linearized pENTR/D-TOPO vector to generate the entry clone using the NEBuilder HiFi DNA Assembly kit (catalogue no. E2621L). To generate the *XVE*>*>axr3-mCherry-KUP5*, *XVE*>*>axr3-mCherry-KUP5m*, *XVE*>*>axr3-mCherry-LRRAC1* and *XVE*>*>axr3-mCherry-LRRAC1m* in *DR5::Luc* background, mutations in KUP5m or LRRAC1m were introduced directly using primers. Cloned sequences were assembled with fragments encoding axr-mCherry and KUP5 or LRRAC1 together with the linearized pENTR/D-TOPO vector to generate the entry clone using the NEBuilder HiFi DNA Assembly Kit (NEB, catalogue no. E2621L). The resulting entry clone was then recombined into the destination vector pMDC7. The specific recombinant fusion sequences of *XVE*>*>axr3-mCherry*, *XVE*>*>axr3-mCherry-KUP5*, *XVE*>*>axr3-mCherry-KUP5m*, *XVE*>*>axr3-mCherry-LRRAC1* and *XVE*>*>axr3-mCherry-LRRAC1m* are provided in Supplementary Table 1. All primers used for plasmid construction are listed in Supplementary Table 2. Final expression constructs were transformed into the *Agrobacterium tumefaciens* strain GV3101 by electroporation. The floral dip method was used to transform *Arabidopsis* plants.

Seeds were surface-sterilized using chlorine gas and sown onto half-strength Murashige and Skoog (1/2 MS) (Biochemie, catalogue no. P14170.01) medium supplemented with 1% (w/v) sucrose and 0.8% (w/v) plant agar, pH 5.9. The seeds were stratified in at 4 °C for 2 days before being grown vertically under long-day photoperiod (16 h light and 8 h dark) at 21 °C. The light sources used were Philips GreenPower light-emitting diode production modules (Philips), combining deep red (660 nm), far red (720 nm) and blue (455 nm), with a photon density of 140.4 µmol m^−2^ s^−1^ ± 3% (ref. ^4^).

### Plant phenotypic assays

For the root formation experiment, sterilized seeds of *XVE*>*>axr3-mCherry*, *XVE*>*>axr3-mCherry-KUP5* or *XVE*>*>axr3-mCherry-KUP5m* were sown directly on 1/2 MS medium containing 1 μM β-oestradiol or Mock and stratified at 4 °C for 2 days. Afterward, they were transferred to normal growth conditions and grown vertically for 5 days. Images were obtained using an Epson scanner (Epson Perfection V800 Photo).

For the root growth rate assay, 4-day-old seedlings were transferred onto 1/2 MS medium containing 1 µM β-oestradiol or Mock, and were then placed onto a vertical flatbed scanner (Epson Perfection V370). AutoIt scripts were used for automatic scanning at 1,200 dots per inch every 2 h as previously described^27^. The generated image series in ImageJ, the StackReg plugin was used for stabilization and manual tracking for root growth measurements.

For lateral root density and root growth assay, 5-day-old seedlings were transferred onto 1/2 MS medium containing 100 nM IAA or 1 µM cvxIAA or 1 µM β-oestradiol or Mock and were grown for a further 4 days. Images were obtained by Epson scanner (Epson Perfection V800 Photo). The lateral root number and main root length were quantified with ImageJ.

For root hair elongation assay, 5-day-old seedlings were transferred onto 1/2 MS solid medium containing 10 nM IAA or 100 nM cvxIAA or 1 µM β-oestradiol or Mock and grown vertically for another 1.5 or 2 days. Images were captured using a stereo microscope (Olympus, catalogue no. SZX16), and root hair length was quantified using ImageJ.

Hypocotyl segment elongation experiment was carried out as described previously^27^. In brief, 3-day-old etiolated seedlings were severed at the apical hook and at the shoot–root conjunction. The resulting hypocotyl segments were placed on a cellophane foil covered with depletion medium containing 10 mM KCl, 1 mM 2-(N-morpholino)ethanesulfonic acid and 1.5% phytagel, pH adjusted to 6.0 with KOH. The samples were left in the dark for 30–60 min before transfer to depletion medium supplemented with 1 µM IAA, or cvxIAA. Scanning was at 16-bit and 1,200 dots per inch every 10 min using the Autolt program. Finally, hypocotyl length was analysed with an ImageJ Macro.

### Protein purification

The coding sequence of ASK1 was cloned into pGEX4T-1 vector. AFB5, IAA7 and IAA17 constructs used for protein expression in this study were published previously^4^. Coding domain sequences of axr2 (P88L) and axr3 (P88L) were cloned into pGEX4T-1 vector, and the resultant plasmids confirmed by sequencing were transformed into *E.* *coli* BL21 competent cells (NEB, catalogue no. C2530H) for protein purification. GST-ASK1 was obtained similarly. Expression and purification of the recombinant GST-tagged proteins from *E.* *coli*, procedure for removal of the GST tag and the use of the ÄKTA start system (GE Healthcare) were all described previously^4^.

### Pull-down assays

In vitro pull-down assays were performed to detect the interaction of TIR1 and its mutated variants with GST-ASK1. The coding sequence of TIR1-Flag was cloned into the pF3A WG (BYDV) Vector (Promega, catalogue no. L5671). Relevant mutated variants of TIR1 were amplified directly from the previously reported plasmids^4^. The corresponding TIR1-Flag and its mutated proteins were obtained through in vitro translation using the TnT SP6 High-Yield Wheat Germ Protein Expression System (Promega, catalogue no. L3260). Pull-down analysis and western blotting were as previously described^4^. Horseradish peroxidase-conjugated FLAG monoclonal antibody (Sigma-Aldrich, catalogue no. A8592) and horseradish peroxidase-conjugated GST monoclonal antibody (Agrisera, catalogue no. AS18 4188) were diluted at 1:2,000 in 5% skim milk for western blotting.

### In vitro AC activity assay and in vivo cAMP measurement

In vitro AC activity assays were performed following our previous publication^4^. For the in vivo cAMP measurement, 5-day-old *XVE*>*>axr3-mCherry* seedlings were first pre-treated with 20 ml of 1/2 MS liquid medium (Mock) or the same medium containing 1 μM β-oestradiol for 4 h to ensure that axr3 is sufficiently induced. Next, the pretreatment medium was replaced with Mock containing 100 nM IAA or 100 nM IAA together with 1 μM β-oestradiol. Root tissues were collected at the indicated time points and were frozen immediately in liquid nitrogen. The method for cAMP isolation and further quantification by liquid chromatography with tandem mass spectrometry has been described previously^4^.

### Confocal microscopy of R2D2 and axr3-AC lines and quantification

The *pTIR1::TIR1*, *pTIR1::ccvTIR1*, *pTIR1::ccvTIR1*^*ACm1*^ and *pTIR1::ccvTIR1*^*ACm3*^ homozygous transgenic lines were crossed with the R2D2 reporter line. The F1 seedlings were used directly for subsequent analysis. Briefly, 4-day-old seedlings were transferred onto solid medium with or without 200 nM cvxIAA and incubated for 1 h. Afterward, the seedlings, together with the medium, were transferred to the chamber (Thermo Fisher Scientific, catalogue no. 155361) and imaged with a vertical Zeiss LSM800 confocal microscope. All parameters were fixed during imaging to enable comparison of R2D2 signals among different genotypes. To quantify the DII-Venus and mDII-tdTomato signals, image analysis was conducted in ImageJ, and the fluorescence intensity of DII-Venus was divided by that of mDII-tdTomato to obtain the DII/mDII ratio. The whole meristem zone was selected for image analysis.

Sterilized seeds of *XVE*>*>axr3-mCherry* or *XVE*>*>axr3-mCherry-KUP5* or *XVE*>*>axr3-mCherry-KUP5m* were germinated on 1/2 MS medium. Four-day-old seedlings were then transferred to medium with 1 µM β-oestradiol. Subsequently, the seedlings, along with the medium, were placed in a chamber (Thermo Fisher Scientific, catalogue no. 155361) and visualized using a vertical Zeiss LSM800 confocal microscope. Imaging parameters were kept consistent throughout the experiment to allow reliable comparisons of mCherry intensity across the different genotypes.

### Luc imaging and quantification

Bioluminescence was imaged and quantified as described^27,28^. The control TIR1 (*pTIR1::TIR1*), *pTIR1::ccvTIR1*, *pTIR1::ccvTIR1*^*ACm1*^ and *pTIR1::ccvTIR1*^*ACm3*^ homozygous transgenic lines were crossed with IAA6, IAA7, IAA8 and IAA17-Luc and DR5::Luc reporter line. Four-day-old F1 seedlings were used for imaging. For steady-state analysis, the seedlings were transferred onto 1/2 MS solid Mock medium or 1/2 MS solid medium containing 200 nM cvxIAA, incubated vertically for 6 h under normal growth conditions and then used for imaging. For timecourse imaging, the seedlings were transferred to 1/2 MS solid medium containing 200 nM cvxIAA using cellophane foil. Then, 1 mM d-luciferin in 1/2 MS liquid medium containing 200 nM cvxIAA or Mock was dropped onto roots before imaging. The seedlings were then live-imaged in a Lumazone Manual Stage Dark Box (Photometric, catalogue no. LMZ-DRK-BOX) and luminescence was captured using a Photometrics Evolve 512 EMCCD camera equipped with a 17-mm fixed lens/0.95 and a second125-mm lens. The multiplier CMCCD gain was set to 300 and 150 for IAA17-Luc and DR5::Luc, respectively. The exposure time was 90 s and the time interval was 2 min.

For DR5::Luc imaging in *XVE*>*>axr3-mCherry-KUP5* or *-KUP5m* and *XVE*>*>axr3-mCherry-LRRAC1* or *-LRRAC1m* background, 4-day-old seedlings were transferred to 1/2 MS medium containing 1 μM β-oestradiol, and 1 mM d-luciferin was applied. Images were taken in the Lumazone Manual Stage Dark Box as described above. The multiplier CMCCD gain was set to 900, the exposure time was 90 s and the time interval was 2 min. Images were analysed in ImageJ, and the whole signal areas of similar width were selected for each root to quantify signal intensity^27,28^.

### Transcriptome sequencing and RT–qPCR

Five-day-old seedlings were transferred into 1/2 MS liquid medium (Mock) or medium containing 200 nM cvxIAA for 3 h. Whole seedlings were collected for RNA extraction, RNA-seq and RT–qPCR analysis. All treatments were conducted with three biological replicates. Transcriptome sequencing services were provided by LEXOGEN (https://www.lexogen.com/). Differential expression analysis use the DESeq2 package^29^. Expression amounts were quantified as fragments per kilobase of transcript per million mapped reads. All genes showing an upregulation with a log_2_ fold change ≥ 1 after 200 nM cvxIAA treatment were selected for heatmap generation using the OmicsStudio platform (https://www.omicstudio.cn/tool/4). The RNA-seq datasets are available from the National Center for Biotechnology Information, under SRA accession numbers SAMN43777066–SAMN43777083. The treatment condition for RT–qPCR in the XVE>>axr3-mCherry transgenic line was consistent with that described for in vivo cAMP measurement. For RT–qPCR assay, RNA extraction was carried out using the RNeasy Plant Mini Kit (Qiagen, catalogue no. 74904). Following the manufacturer’s instructions of the RevertAid First Strand cDNA Synthesis Kit (Thermo, catalogue no. K1622), genomic DNA was removed, and 1 μg of total RNA was used for reverse transcription. The cDNA was diluted 20-fold before qPCR. Samples were prepared in three technical replicates using the Automated workstation Biomek i5 (Beckman Coulter). qPCR was performed using the LightCycler 480 (Roche) with Luna Universal rtPCR Master Mix (NEB, catalogue no. M3003S). The sequences of gene-specific primers used are listed in Supplementary Table 2. Relative gene expression was calculated using the ΔΔCT method with PROTEIN PHOSPHATASE 2A SUBUNIT A3 (PP2A) as the reference gene.

### Software and statistical analysis

All graphs were generated using GraphPad Prism v.8.0.1. Figures were prepared with Adobe Illustrator 2021. One-way ANOVA, two-way ANOVA, *t*-test and multiple comparisons were performed using GraphPad Prism v.10.2.2.

### Reporting summary

Further information on research design is available in the Nature Portfolio Reporting Summary linked to this article.

## Online content

Any methods, additional references, Nature Portfolio reporting summaries, source data, extended data, supplementary information, acknowledgements, peer review information; details of author contributions and competing interests; and statements of data and code availability are available at 10.1038/s41586-025-08669-w.

## Supplementary information

Supplementary Information 1 RNA-seq, gene expression levels and differential expression analysis in this study.

Reporting Summary

Supplementary Table 1 The recombinant sequences of XVE>>axr3-mCherry and XVE>>axr3-mCherry-LRRAC1/LRRAC1m/ KUP5/ KUP5m.

Supplementary Table 2 All primers used in this study.

## Source data

Source Data Fig. 1

Source Data Fig. 2

Source Data Fig. 3

Source Data Fig. 4

Source Data Extended Data Fig. 1

Source Data Extended Data Fig. 2

Source Data Extended Data Fig. 3

Source Data Extended Data Fig. 4

Source Data Extended Data Fig. 5

Source Data Extended Data Fig. 6

Source Data Extended Data Fig. 7

## Data Availability

Data supporting the findings of this study are available in the paper. The RNA-seq datasets are available from the NCBI under SRA accession numbers SAMN43777066–SAMN43777083. Source data are provided with this paper.

## Code availability

No custom codes were developed in this study.
