# Supplementary material for: TIR1-produced cAMP as a second messenger in transcriptional auxin signalling
Source: Nature. 2025 Mar 5;640(8060):1011–6. doi: 10.1038/s41586-025-08669-w (PMC12018254; doi:10.1038/s41586-025-08669-w)
Supplement: Supplementary file 2 — Reporting Summary [file 41586_2025_8669_MOESM2_ESM.pdf]

nature portfolio

Corresponding author(s): Jiří Friml

Last updated by author(s): Jan 14, 2025

Reporting Summary

Nature Portfolio wishes to improve the reproducibility of the work that we publish. This form provides structure for consistency and transparency in reporting. For further information on Nature Portfolio policies, see our Editorial Policies and the Editorial Policy Checklist.

Statistics

For all statistical analyses, confirm that the following items are present in the figure legend, table legend, main text, or Methods section.

n/a	Confirmed	
☐	☒	The exact sample size (*n*) for each experimental group/condition, given as a discrete number and unit of measurement
☐	☒	A statement on whether measurements were taken from distinct samples or whether the same sample was measured repeatedly
☐	☒	The statistical test(s) used AND whether they are one- or two-sided<br>*Only common tests should be described solely by name; describe more complex techniques in the Methods section.*
☒	☐	A description of all covariates tested
☒	☐	A description of any assumptions or corrections, such as tests of normality and adjustment for multiple comparisons
☐	☒	A full description of the statistical parameters including central tendency (e.g. means) or other basic estimates (e.g. regression coefficient) AND variation (e.g. standard deviation) or associated estimates of uncertainty (e.g. confidence intervals)
☐	☒	For null hypothesis testing, the test statistic (e.g. *F*, *t*, *r*) with confidence intervals, effect sizes, degrees of freedom and *P* value noted<br>*Give P values as exact values whenever suitable.*
☒	☐	For Bayesian analysis, information on the choice of priors and Markov chain Monte Carlo settings
☒	☐	For hierarchical and complex designs, identification of the appropriate level for tests and full reporting of outcomes
☒	☐	Estimates of effect sizes (e.g. Cohen's *d*, Pearson's *r*), indicating how they were calculated

Our web collection on statistics for biologists contains articles on many of the points above.

Software and code

Policy information about availability of computer code

Data collection	The microscope images were collected using ZEN 2011 (blue edition)
Data analysis	Root length was measured using the segmentation plugin Simple Neurite Tracer in Image J (ImageJ bundled with 64-bit Java 8 for windows, DOI: 10.1093/bioinformatics/btr390);<br>GraphPad Prism 10.2.2 and 8.0.1 were used for graph preparation and statistical analysis.<br>The differential expression analysis of RNA-Seq data was performed with DESeq2 package.<br>The figure panels were designed using Adobe Illustrator 2021.

For manuscripts utilizing custom algorithms or software that are central to the research but not yet described in published literature, software must be made available to editors and reviewers. We strongly encourage code deposition in a community repository (e.g. GitHub). See the Nature Portfolio guidelines for submitting code & software for further information.

Data

Policy information about availability of data

All manuscripts must include a data availability statement. This statement should provide the following information, where applicable:

- Accession codes, unique identifiers, or web links for publicly available datasets
- A description of any restrictions on data availability
- For clinical datasets or third party data, please ensure that the statement adheres to our policy

All data generated or analysed during this study are included in this published article. The RNA-seq datasets are available from the National Center for Biotechnology Information, under SRA accession numbers SAMN43777066-SAMN43777083.

Research involving human participants, their data, or biological material

Policy information about studies with human participants or human data. See also policy information about sex, gender (identity/presentation), and sexual orientation and race, ethnicity and racism.

Reporting on sex and gender	N/A
Reporting on race, ethnicity, or other socially relevant groupings	N/A
Population characteristics	N/A
Recruitment	N/A
Ethics oversight	N/A

Note that full information on the approval of the study protocol must also be provided in the manuscript.

Field-specific reporting

Please select the one below that is the best fit for your research. If you are not sure, read the appropriate sections before making your selection.

☒ Life sciences ☐ Behavioural & social sciences ☐ Ecological, evolutionary & environmental sciences

For a reference copy of the document with all sections, see nature.com/documents/nr-reporting-summary-flat.pdf

Life sciences study design

All studies must disclose on these points even when the disclosure is negative.

Sample size	No sample-size calculation was performed. Sample sizes were chosen according to experience or preliminary tests.
Data exclusions	No data were excluded from the analysis.
Replication	All the data were repeated independently at least twice. All the experimental findings were reproducible.
Randomization	Sample allocation was random.
Blinding	The analysis was performed blindly wherever possible. For those cases in which blinding was not possible, because sample allocation, data collection and data analysis were done by the same investigator, we guarantee that all of them were done in a strictly objective way.

Reporting for specific materials, systems and methods

We require information from authors about some types of materials, experimental systems and methods used in many studies. Here, indicate whether each material, system or method listed is relevant to your study. If you are not sure if a list item applies to your research, read the appropriate section before selecting a response.

Materials & experimental systems

n/a	Involved in the study	
☐	☒	Antibodies
☒	☐	Eukaryotic cell lines
☒	☐	Palaeontology and archaeology
☒	☐	Animals and other organisms
☒	☐	Clinical data
☒	☐	Dual use research of concern
☐	☒	Plants

Methods

n/a	Involved in the study	
☒	☐	ChIP-seq
☒	☐	Flow cytometry
☒	☐	MRI-based neuroimaging

Antibodies

Antibodies used	Anti-GST-tag, HRP-conjugated monoclonal antibody (Agrisera, AS18 4188);Anti-FLAG-Peroxidase (Sigma, SLCF0816).
Validation	The antibodies were used for western blot experiments. They are commercially available, validated by the producers, and routinely used in many labs.

Plants

Seed stocks	The Arabidopsis seeds used in the study are all previously published and appropriately cited or acknowledged in the manuscript
Novel plant genotypes	Does not involve novel plant genotypes.
Authentication	The transgenic seeds were either generated in our group or provided by the authors of publication. And no authentication was done by the authors.
